# Correlations between the Frankfort Plane and the Presence of Myofascial Trigger Points in Posterior Cervical Musculature: An Exploratory Study

**DOI:** 10.3390/jcm13123614

**Published:** 2024-06-20

**Authors:** Darío Sánchez-Guilabert, Ángel Martínez-Carrasco

**Affiliations:** Department of Physiotherapy, Campus de Ciencias de la Salud, University of Murcia, 30120 Murcia, Spain; dario.sanchezg@um.es

**Keywords:** neck pain, trigger points, myofascial pain syndromes, posture, cephalometry, photogrammetry

## Abstract

Neck pain is a pathology with a high impact in terms of physical disability in modern society. The position of the head is related to neck pain. The Frankfort plane determines the position of the skull in space. The profile photograph of the subjects was used to determine the Frankfort plane and to study its degree of inclination. Myofascial pain syndrome is one of the most common causes of musculoskeletal pain. Trigger points are hyperirritable spots located in a palpable taut band of skeletal muscle that is painful on compression or stretch and causes a local twitch in response to snapping or palpation of the band. **Objectives:** The aim of this study was to analyze the relationship between the Frankfort plane and the presence of myofascial trigger points causing cervical myofascial pain. **Methods**: This is a cross-sectional descriptive observational study. All subjects underwent a photographic study to determine the degree of Frankfort plane inclination, and the posterior cervical musculature was palpated to find myofascial trigger points that were measured with a pressure algometer in three cervical locations on the right and left sides. **Results:** Our study included 47 subjects who had suffered at least one episode of cervical pain in their lifetimes. The mean age was 22.3 ± 2.9 years. Statistically significant results were found in the first right location and sports practice (*p* = 0.007), in the second right location and gender (*p* = 0.0097), in the second right location and sports practice (*p* = 0.0486), in the third right location and gender (*p* = 0.0098), and in the first, second, and third left locations and gender (*p* = 0.0083; *p* = 0.024; *p* = 0.0016, respectively). In the correlation between the Frankfort plane and the presence of myofascial trigger points, all locations were positive, with the first right location being statistically significant (*p* = 0.048). **Conclusions:** A positive relationship was found between the Frankfort plane and the presence of myofascial trigger points. The greater the angle of the Frankfort plane, the less the myofascial pain.

## 1. Introduction

Neck pain is increasingly common in the current population. It is a highly disabling pathology that affects the subject not only at work, but also in the family environment. The annual worldwide incidence of neck pain ranges from 10.4% to 21.3% [1]. In Spain, the neck is the second most frequent site of pain, with a higher prevalence in females (25.68%) than in males (12.54%). The prevalence of neck pain increased from 2014 to 2019 in both males and females [2].

Neck pain might present with different symptoms, but the most common is usually classified as myofascial pain syndrome (MPS), one of the most common causes of musculoskeletal pain [3], representing 71% of medical consultation in primary healthcare [4]. MPS is defined as pain of muscular origin, characterized by the presence of myofascial trigger points (MTrPs), decreased ability of motion, sleep disorders, and a referred pain pattern. MTrPs are hyperirritable spots located in a palpable taut band of skeletal muscle that is painful on compression or stretch and causes a local twitch in response to snapping or palpation of the band [5,6]. 

To diagnose the presence of MTrPs, some criteria must be met: the presence of a palpable tight band in the muscle, the presence of a stiff and hyperirritable point within a tight band, reproduction of the patient’s symptomatology on palpation of the stiff point, referred pain pattern caused by compression of the point, and a local spasm response caused by stimulation of the point by compression or dry needling. 

MTrPs can be active or latent depending on clinical characteristics. An active trigger point has an area of tenderness during rest or causes spontaneous pain on palpation. A latent trigger point may show hypersensitivity on palpation, but does not spontaneously cause pain [7,8,9].

The etiology of MPS is currently unknown, but it is known that the causes are related to biomechanical factors of overload, muscle overuse, or repetitive microtrauma in which the function of the motor plate is altered. Some of the currently known factors activating MTrPs are acute trauma, repeated microtrauma, lack of exercise (muscle weakness), muscle strains, poor posture, vitamin deficiency, sleep disorders, and worn joints [10,11]. Due to the characteristics of this study, special attention will be paid to postural alterations that lead to the formation of MTrPs in the back of the neck.

Posture is defined as the position that the body adopts in the space where it is located, governed by the physical law of equilibrium. A body is in equilibrium when the vertical line of the center of gravity falls in the center of the support base. This vertical line coincides with the midline of the body in the frontal plane. In the sagittal plane, the center of gravity is slightly in front of the fourth lumbar vertebra, and the line of gravity passes slightly in front of the tibiotalar joint, through the glenumeral joint, and through the ear lobe [12].

In recent years, there has been an increase in the prevalence of neck pain; one of the causes of this is the overuse of technology, such as too many hours hunched over a computer or a smartphone, which often triggers muscle strain, causes pain, and restricts motion [13]. In a study conducted on university students, it was observed that excessive use of computers or activities requiring concentration led students to adopt a forward head position, resulting in the development of neck pain [14]. The forward posture of the head, together with other postural alterations, is included in what is known as upper-crossed syndrome (UCS). The posterior cervical musculature has an antigravity function that keeps the head upright when standing or sitting. The activation of this musculature only appears during activities that alter the balance of the head, such as a forward head position. The imbalance produced leads to increased tension in the posterior neck muscles, thus producing pain [15,16]. 

The Frankfort horizontal plane was introduced in 1872 by Professor Von Ihering. It was finally accepted at the 13th General Congress of the German Anthropological Society in 1882 in the German city of Frankfort-on-Main. The Frankfort plane (FP) was developed to standardize a reference plane for the study of the skull by drawing a horizontal line connecting the anatomical porion (PoA), the highest point on the bony outline of the external auditory meatus, and the orbitale (Or), the lowest possible point on the bony right or left orbital rim [17]. 

Originally, the FP was developed to join two references located on the bone surface, but in different disciplines, references located on the skin surface are used, resulting in the plane that joins the highest point of the tragus on the ear (external auditory canal) to the lowest possible point of the orbital orifice on the skin [18]. One of the advantages offered by the FP is that by orienting the skull so that the FP coincides with the horizontal direction, the skull adopts a position similar to the one it had when it was alive and attached to the spine. In living subjects, the position of the head is not always aligned with the FP in the horizontal direction, as the tension of the neck muscles may vary its angle towards head flexion (negative FP) or head extension (positive FP), the natural position of the head being different for each person. The FP is also used in the study of posture, muscle chains, and global postural re-education (GPR) [19,20,21].

In the field of craniofacial measurements, two methods are used: cephalometry and photogrammetry. Cephalometry is a study carried out on the basis of craniofacial measurements obtained from tracings made on standardized lateral skull radiographs. Photogrammetry corresponds to the analysis of anthropometric distances on a lateral photograph. In facial photogrammetry, the standardization of the position of the head is fundamental. There are protocols that endorse the natural head position (NHP) as the initial state to take the photograph of the subject. The FP can be practically coincident in clinical radiographs and clinical photographs by applying a systematized protocol for photographic registration and for obtaining NHP [22,23,24,25]. 

Published studies on the use of the Frankfort plane to determine head position are exclusively in the fields of dentistry and anthropometry. In our study, the FP is used in a novel way in the field of physiotherapy, and its relationship to MTrPs causing neck pain is analyzed. The main objective of this work is to determine the relationship between head tilt, using FP measurement, and the presence of pain caused by the presence of MTrPs in the posterior neck musculature. The secondary purpose of this work is to bring the use of digital photography and cephalometry to the field of physiotherapy, since the advance of new technologies is facilitating research and evaluation in physiotherapy. The hypotheses of this study are that there is a relationship between the degree of head inclination measured by FP and the presence of neck pain caused by MTrPs, and that there are factors that influence this relationship, such as gender, the practice of sports, the use of electronic devices, and hours of study.

## 2. Materials and Methods

### 2.1. Study Design

This work is a cross-sectional descriptive observational study in which the Frankfort plane was analyzed photographically, and the presence of myofascial trigger points was analyzed by palpation and algometer measurement in the posterior cervical musculature.

In the first phase of the study, a literature search was conducted to analyze published articles on FP and MTrPs. In the second phase, the study and the intervention were designed and subsequently supervised by a methodology expert from the Scientific and Technical Research Area (ACTI) of the University of Murcia. In the third phase of the study, fourth-degree physiotherapy students were contacted, and the convenience sampling method was used. In the fourth phase of the study, the physiotherapist who performed the measurements was instructed, and the intervention was carried out over five days. Finally, a statistical analysis of the data obtained was performed. The subjects analyzed were unaware of the purpose of the study.

The study adhered to the Helsinki Declaration. It was approved by the Research Ethics Committee of the University of Murcia under code 3358/2021.

### 2.2. Participants

The study was conducted on a sample of fourth-degree physiotherapy students at the University of Murcia. The subjects who took part in the sample did so voluntarily after reading and signing the previous consent document before the start of the study. Out of the 78 students who took part in the course, 47 met the inclusion and exclusion criteria and agreed to participate in this study.

The inclusion criteria were that participants were university students in the fourth year of a physiotherapy degree, with an age range between 18 and 40 years, who had suffered cervical pain at least once in their lives, who spent several hours a day in front of a screen at work or for leisure, and who agreed to participate in the study by signing the consent document. The exclusion criteria disqualified anybody with a neurological disease, having suffered a recent injury or trauma in the cervical area, with scoliosis or any other relevant spine disease, or having any musculoskeletal pathology that could affect the results of the study or prevent the person from maintaining a stable posture.

**Figure 1:**
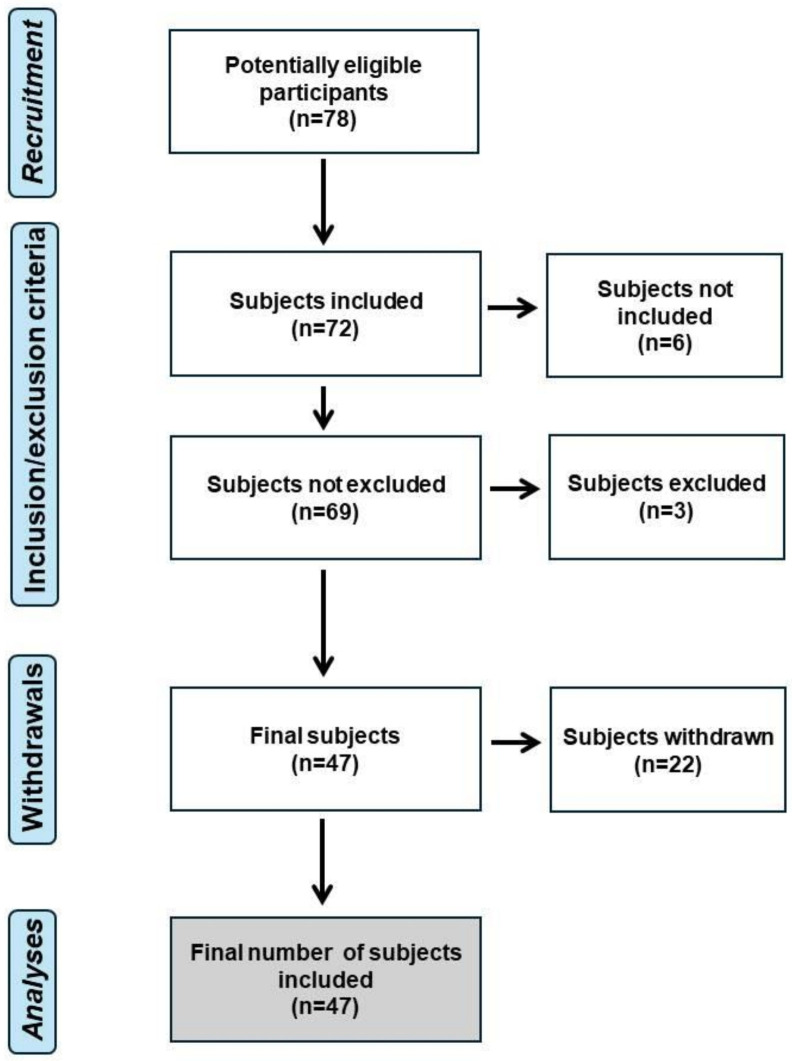
Flowchart of the study.

Six subjects were not included because they had not suffered neck pain at least once in their lives. Two subjects were excluded for having scoliosis, and one for having suffered a recent trauma to the cervical area. Twenty-two subjects declined to continue in the study when they learned that they had to have a profile picture taken, which reduced the sample size considerably (Figure 1).

### 2.3. Intervention

To calculate the degrees of FP inclination of the participants, a photographic study of the profile of each subject was carried out, and then the degrees of inclination were calculated using the Adobe Photoshop CC 2019 program licensed by the University of Murcia.

**Figure 2:**
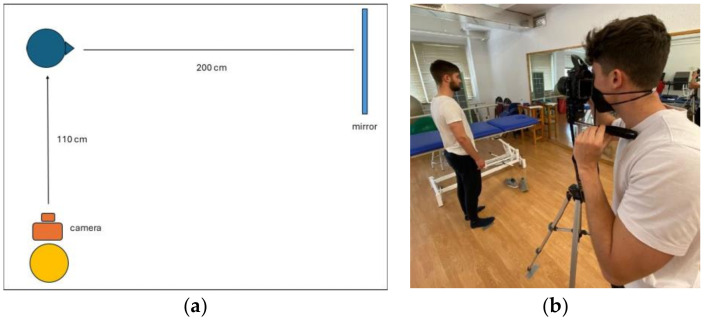
(**a**,**b**) Photographic study of the Frankfort plane.

Before taking the photograph, the participants were asked to remove things that could interfere with the measurements, such as earrings, eyeglasses, and masks. The lowest possible point of the right orbital orifice was then located by palpation and marked with a black Staedtler Lumocolor permanent marker. The camera was placed on a tripod equipped with a bubble level adjusted to the height of the subject’s head and focusing on the face 110 cm from the subject, who was placed in front of a mirror 200 cm away (Figure 2).

In order to obtain a reliable reference of the horizontal line, the virtual horizon electronic gyroscope incorporated in the camera was used. To take the photograph, the subjects were placed in profile at the indicated location, and the protocol proposed by Solow and Tallgren was used to achieve the natural head position (NHP). To achieve the NHP before taking the photograph, the standing subject was asked to take a few steps without moving off the examination site to promote relaxation. Subsequently, the subject was asked to perform full flexion and extension movements of the cervical spine in a decreasing manner until the head was in a self-balancing position. The subject was then asked to look at their reflection in the mirror and finally to swallow saliva. Two seconds later, the photograph was taken [23]. 

The camera used was a Nikon D610 full-format FX camera in manual mode, ISO 100, aperture f11, shutter speed 1/125 s, with a 105 mm lens and built-in flash.

**Figure 3:**
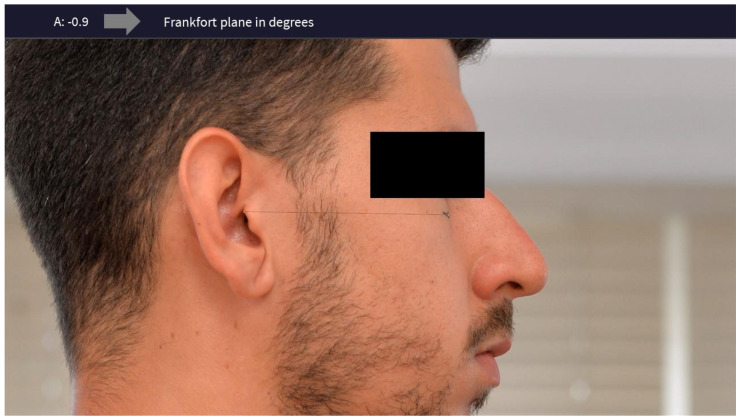
Frankfort plane in degrees using Adobe Photoshop.

After taking the photos, a line was drawn using Adobe Photoshop between the two reference points to determine the FP in the digital image of each of the subjects. The Photoshop ruler tool was used to obtain the degree of inclination of the FP (Figure 3). In the study by Devi et al., to determine the reliability of the true horizontal plane (THP) with the Frankfort horizontal plane (FHP), the measurements obtained were subjected to statistical analysis by an independent *t*-Test (*p* < 0.05). The statistical results of the parameters showed insignificant differences, indicating that the FHP is as reliable as the THP [26]. 

To determine if any pain was experienced, the presence of taut bands and MTrPs in the posterior cervical musculature were analyzed by flat palpation of the right and left posterior neck musculature in three locations. First, the muscular insertion of the semispinalis cervicis or semispinalis colli at the base of the skull was palpated, then the middle zone was palpated, and finally, the lower muscular zone of the semispinalis cervicis was palpated. 

**Figure 4:**
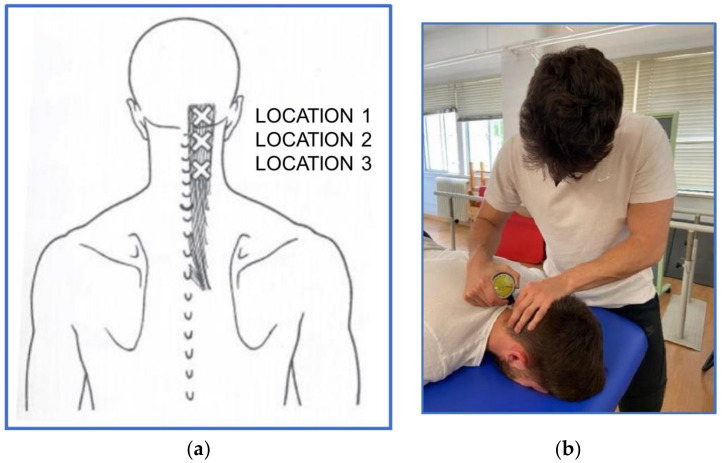
(**a**) MTrPs locations [16]. (**b**) Measurement with analog algometer.

In areas where tension bands and MTrPs were detected, they were measured using a pressure algometer until the pressure pain threshold (PPT) was reached. The PPT is defined as the point at which a non-painful pressure stimulus becomes a painful pressure sensation. In the study by Chesterton et al., it was determined that the reliability for measuring PPT was not significantly different between the values. The intraclass correlation (ICC) was 0.91, with a confidence interval (CI) of 95% CI 0.82, 0.97 [27]. In the study by Kinser et al., the validity of the pressure algometer compared to a force platform was found to be excellent, with mean Pearson’s correlations (r = 0.990) for maximal force and (r = 0.999) for increased forces [28]. The algometer used was a Wagner Force Dial^®^ Fdk 20 (Wagner instrument, Greenwich, CT, USA) that measures from 0 to 10 kg. Once the MTrPs are located, the tip of the algometer is placed perpendicular to the muscle, and the pressure is increased at a rate of 1 kg/second until the subject experiences pain and alerts the evaluator [29,30,31] (Figure 4). 

### 2.4. Data Collection

All researchers involved in data collection were trained in and practiced standardized data collection and measurement procedures. 

Sociodemographic data (gender and age) were initially collected. Participants filled in a form and were asked for the approximate time spent using electronic devices per day, the approximate number of hours of study per day, if they played sport at least three times a week, if they had suffered from neck pain in the past year, and if they had suffered from neck pain in the last fifteen days. 

Profile photographs were taken to determine the degree of FP using Adobe Photoshop. 

To measure the PPT, the MTrPs were scanned with an analog algometer at locations 1, 2, and 3 on the right and left sides in the posterior neck musculature. 

### 2.5. Statistical Analysis

For data management, a coding process was carried out for statistical analysis. A database was created in which the values of the measurements and the different sociodemographic variables were entered.

The IBM SPSS v.24 (Statistical Package for the Social Sciences, IBM Corp, Armonk, NY, USA) statistical program was used to create the database.

Data analysis was performed by the Statistical Support Section (SAE) of the Scientific and Technical Research Area (ACTI) of the University of Murcia using R v4.0.3 (R Core Team 2020).

The study consisted of a descriptive analysis of the sample, a cross-analysis between two variables (FP and PPT), together with the different sociodemographic variables and the questions of the survey. Finally, a correlation between FP and PPT was determined. A Shapiro–Wilk test and a Barlett test were performed to check for normality and homoscedasticity of the variables. An alpha value of 0.05 was set across all the analyses performed.

## 3. Results

The final sample consisted of 47 participants, ranging in age from 21 to 38 years old. Table 1 summarizes the baseline demographic data of the 47 subjects included, along with the results.

In the descriptive analysis of FP, a positive value indicates that the head is in extension, while a negative FP value indicates that the head is in flexion. The minimum value obtained was −5° and the maximum value was 14.4°, with a mean of 6.05 ± 4.6°. Most of the subjects in the sample had an FP in extension. The FP in women achieved a mean of 5.40 ± 4.13°, while in men it was 6.54 ± 4.94°.

The PPT was measured in kg/cm^2^ at locations 1, 2, and 3 on the right side and on the left side. The first right location had a mean of 2.69 ± 1.71 kg/cm^2^. The second right location had a mean of 2.47 ± 1.48 kg/cm^2^. The third right location had a mean of 3.07 ± 1.96 kg/cm^2^. The first left location had a mean of 2.06 ± 0.91 kg/cm^2^. The second left location had a mean of 2.34 ± 1.4 kg/cm^2^. The third left location had a mean of 2.51 ± 1.38 kg/cm^2^.

Table 2 shows the results of the cross-analysis of FP, PPT, and the different study variables. When there was normality and homoscedasticity, an independent *t*-Test was performed. If there was no normality but homoscedasticity, a Mann–Whitney U Test (nonparametric test) was performed. If there was neither normality nor homoscedasticity, a *t*-Test with Welch’s correction was performed.

Statistically significant results were found in the first right location and the practice of sports at least three times a week (*p* = 0.007); the subjects practicing sports had a higher PPT (less myofascial neck pain). It was found that there were significant differences between the second right location and gender (*p* = 0.0097), with males presenting a higher PPT (less pain) than females. In the second right location and the practice of sports at least three times a week, significant differences were found (*p* = 0.0486); again, the study subjects who practiced sports had a greater PPT (less pain). In the third right location and gender (*p* = 0.0098), males had a higher PPT than females (less pain). In the first, second, and third left locations (*p* = 0.0083; *p* = 0.024; *p* = 0.0016), males had a higher PPT than females (less pain).

**Figure 5:**
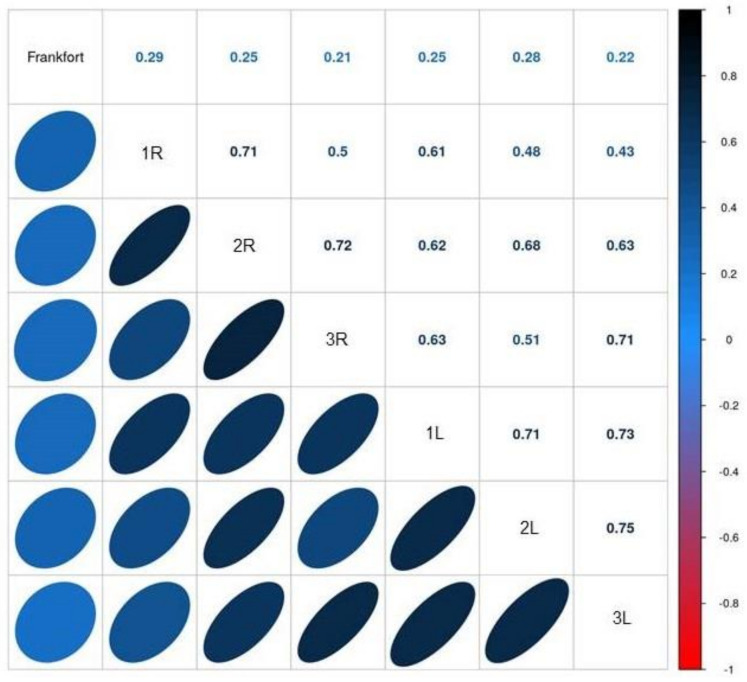
Spearman correlation coefficients for Frankfort plane and pressure pain threshold associations with continuous results. Interpretation of the correlation matrix: Each row-column pair represents a correlation of the two variables; all correlations were positive and are shown in blue. The color intensity is proportional to the correlation coefficients: 0 = no correlation, −1 = perfect inverse correlation, and 1 = perfect direct correlation.

Figure 5 shows the Spearman correlation coefficients for the associations of the Frankfort plane and pressure pain threshold at locations 1, 2, and 3 with continuous results. All correlations were positive. FP was directly associated with right location 1 PPT (r = 0.29, *p* = 0.048). PF was positively associated with PPT at location 2 right (r = 0.25, *p* = 0.084). FP was associated with PPT at location 3 right (r = 0.21, *p* = 0.148). FP was associated with PPT at location 1 left (r = 0.25, *p* = 0.094). FP was associated with PPT at left location 2 (r = 0.28, *p* = 0.055). FP was associated with PPT at location 3 left (r = 0.22, *p* = 0.137).

## 4. Discussion

Cephalometric studies are widely used in other branches of health care, such as dentistry and maxillofacial surgery, in addition to the evaluation of facial growth and development. In the field of physiotherapy, there are very few articles describing the relationship between cranial references, such as FP, and the presence of posture-related dysfunctions or pathologies. 

Some studies relate postural alterations to myofascial pain syndrome, characterized by the presence of MTrPs [10,11]. Cervical pain is a very frequent pathology, and the common form of presentation is classified as myofascial pain syndrome [3,4]. The study by Jiménez-Trujillo [2] showed a higher prevalence of cervical pain in women than in men. The same is shown in the study by Capó-Juan [3], in which women had a higher prevalence of neck pain than men in the sample. Our study coincides with these two authors, since there were statistically significant differences when measuring the PPTs between men and women, with significantly greater cervical pain in women than in men.

In our study, the relationship between the practice of sports at least three times a week and the presence of cervical pain caused by MTrPs has been proved. There were significant differences between subjects who practiced sports and those who did not. This was described in the study carried out by Díaz [7], in which it was indicated that the lack of physical activity, together with a sedentary lifestyle, can produce a chronic muscular imbalance in which the postural muscles are tense and the dynamic muscles are inhibited. This situation is a predisposing factor to suffering cervical myofascial pain. Yap EC [32] stated that a sedentary lifestyle can produce an imbalance in which the static muscles that maintain posture become increasingly stiff and tense.

Another risk factor for the development of myofascial neck pain is the increasing use of electronic devices, such as computers and telephones. In the study by Kang et al. [13], a group of workers who spent more than six hours on a computer were found to be more prone to neck pain, with the head anteriorized and the cervical spine in hyperextension. This does not coincide with the results obtained in our study, since there were no significant differences in the relationship between pain and the time spent using electronic devices.

In a study conducted by Pacheco et al. [14], the sample was composed of university students whose PPTs were measured to relate them to the forward position of the head. This study suggested that there is no relationship between forward head position and its association with the PPT in asymptomatic college students, whereas in students with subclinical neck pain, increased forward head position was associated with right trapezius hypoalgesia and shorter duration of neck pain. These findings are inconsistent with current hypotheses on the association between neck pain and forward head position. In our study, we also did not obtain significant results when relating time spent studying and using electronic devices with neck pain.

In the Rubine-Gatina study [33], it was observed that the sternocleidomastoid muscle, whose action is neck flexion in addition to supporting the head, can cause head and neck pain due to the prolonged use of computers while teleworking. The study determined that the minimum tension of the sternocleidomastoid muscle occurs when the head is placed in flexion at angles between −30° and −60°. In our study, the posterior neck musculature whose function is neck extension was assessed, and it was determined that the greater the neck extension, the less pain is observed. 

According to Travell and Simons [16], this misalignment of head balance over time results in increased tension in the cervical muscles and supporting structures, causing a propensity for cervical injury and pain. Slight flexion of the head and neck increases the tightness of the taut bands and the pressure sensitivity of the MTrPs of the posterior cervical muscles. The semispinalis cervicis muscle has the main action of head extension, also working in the antigravity control of the head when the subject leans forward. Postural stress, such as reading or working at a desk seated with the head in a forward position or with the neck in maintained flexion, commonly activates and perpetuates posterior cervical MTrPs. This is reflected in our study, as the subjects who had the head in flexion position with a lesser degree of FP presented greater pain on pressure (PPT).

Our study has several limitations, such as the small sample size, the fact that it was conducted with a sample of university students aged between 21 and 38 years, and the fact that it has an observational design, which limit the strength and external validity of our results. There is a need for further research studies that include a larger number of subjects and compare the relationship between forward head position, FP, and posterior neck pain produced by MTrPs.

## 5. Conclusions

The results derived from our study highlight that a positive relationship has been observed between the Frankfort plane and the presence of myofascial trigger points. The main conclusions were that the greater the Frankfort plane angle, the lesser the myofascial pain, and the lesser the Frankfort plane angle, the greater the myofascial pain.

Females have a lower pressure pain threshold than males, and therefore have more predisposition to suffer cervical pain of myofascial origin.

Subjects who practice sports at least three times a week have a higher pressure pain threshold and therefore suffer less neck pain.

Excessive use of electronic devices and study hours were not related to a greater presence of neck pain.

The results of this study can be used in clinical practice in the evaluation and treatment of patients with neck pain because there is a positive relationship between the Frankfort plane and the presence of myofascial trigger points in the posterior cervical musculature. In addition, regular physical exercise may be indicated to reduce neck pain caused by myofascial trigger points.

In the future, it would be interesting to confirm through clinical trials whether changes in the Frankfort plane inclination occur after the application of physiotherapy treatment in patients with neck pain.

## Figures and Tables

**Table 1:** Population, characteristics, and outcomes.

Variable	Value (N = 47)
Age (years)	22.3 ± 2.9
Females (n, %)	20 (42.5%)
Males (n, %)	27 (57.5%)
Time using electronic devices	
Less than three hours	2 (4.3%)
Three to six hours	33 (70.2%)
More than six hours	12 (25.5%)
Number of hours of study per day	
No time	3 (6.4%)
One hour	12 (25.5%)
Two hours	22 (46.8%)
Three hours	5 (10.6%)
Four hours	4 (8.5%)
Six hours	1 (2.1%)
Sport at least three times a week	
Yes	38 (80.9%)
No	9 (19.1%)
Have you had neck pain in the last year?	
Yes	36 (76.6%)
No	11 (23.4%)
Have you had neck pain in the last fifteen days?	
Yes	27 (57.4%)
No	20 (42.6%)

**Table 2:** Associations between FP, PPT, and the different variables of the study.

Associations	Test Value	*p*-Value
FP/Gender	Independent *t*-Test = 0.858	0.395
FP/Time electronic devices	Independent *t*-Test = 0.413	0.685
FP/Hours of study	Independent *t*-Test = −1.135	0.268
FP/Sport	Independent *t*-Test = 0.441	0.666
FP/Neck pain in the last year	Independent *t*-Test = 0.091	0.091
FP/Neck pain last 15 days	Independent *t*-Test = −1.044	0.302
PPT 1R/Gender	Mann-Whitney U Test = 356	0.065
PPT 1R/Time e. Devices	Welch’s *t*-Test = −1.454	0.168
PPT 1R/Hours of study	Mann-Whitney U Test = 180	0.174
PPT 1R/Sport	Welch’s *t*-Test = 2.798	**0.007**
PPT 1R/pain last year	Mann-Whitney U Test = 252	0.178
PPT 1R/Pain last 15 days	Mann-Whitney U Test = 271	0.991
PPT 2R/Gender	Mann-Whitney U Test = 390.5	**0.0097**
PPT 2R/Time e. Devices	Welch’s *t*-Test = −1.019	0.325
PPT 2R/Hours of study	Welch’s *t*-Test = −1.510	0.138
PPT 2R/Sport	Welch’s *t*-Test = −2.029	**0.0486**
PPT 2R/pain last year	Welch’s *t*-Test = −1.157	0.254
PPT 2R/Pain last 15 days	Mann-Whitney U Test = 311	0.383
PPT 3R/Gender	Mann-Whitney U Test = 390	**0.0098**
PPT 3R/Time e. Devices	Mann-Whitney U Test = 212	0.971
PPT 3R/Hours of study	Mann-Whitney U Test = 200.5	0.099
PPT 3R/Sport	Mann-Whitney U Test = 145	0.489
PPT 3R/pain last year	Mann-Whitney U Test = 266	0.088
PPT 3R/Pain last 15 days	Mann-Whitney U Test = 336	0.157
PPT 1L/Gender	Welch’s *t*-Test = 2.803	**0.0083**
PPT 1L/Time e. Devices	Mann-Whitney U Test = 182.5	0.508
PPT 1L/Hours of study	Welch’s *t*-Test = −1.108	0.273
PPT 1L/Sport	Welch’s *t*-Test = −0.839	0.407
PPT 1L/pain last year	Welch’s *t*-Test = 0.991	0.343
PPT 1L/Pain last 15 days	Welch’s *t*-Test = 0.449	0.656
PPT 2L/Gender	Mann-Whitney U Test = 375	**0.024**
PPT 2L/Time e. Devices	Mann-Whitney U Test = 171	0.346
PPT 2L/Hours of study	Mann-Whitney U Test = 237	0.954
PPT 2L/Sport	Mann-Whitney U Test = 171	0.346
PPT 2L/pain last year	Mann-Whitney U Test = 193.5	0.561
PPT 2L/Pain last 15 days	Mann-Whitney U Test = 304.5	0.463
PPT 3L/Gender	Mann-Whitney U Test = 417	**0.0016**
PPT 3L/Time e. Devices	Mann-Whitney U Test = 194	0.705
PPT 3L/Hours of study	Welch’s *t*-Test= −0.462	0.648
PPT 3L/Sport	Welch’s *t*-Test= −1.309	0.198
PPT 3L/pain last year	Welch’s *t*-Test= 1.228	0.243
PPT 3L/Pain last 15 days	Mann-Whitney U Test = 305	0.457

Abbreviations: FP, Frankfort plane; PPT, pressure pain threshold; Locations 1, 2, and 3; R, right, L, left. The numbering in bold indicates that they are statistically significant.

## Data Availability

The data presented in this study are available on request from the corresponding author. The data are not publicly available due to privacy issues.

## References

[1] Hoy D.G., Protani M., De R., Buchbinder R. (2010). The Epidemiology of Neck Pain. Best Pract. Res. Clin. Rheumatol..

[2] Jiménez-Trujillo I., López-de-Andrés A., Del Barrio J.L., Hernández-Barrera V., Valero-de-Bernabé M., Jiménez-García R. (2019). Gender Differences in the Prevalence and Characteristics of Pain in Spain: Report from a Population-Based Study. Pain Med. Malden Mass.

[3] Capó-Juan M.A. (2015). Cervical myofascial pain syndrome. Narrative review of physiotherapeutic treatment. An. Sist. Sanit. Navar..

[4] Calsina-Berna A., Moreno Millán N., González-Barboteo J., Solsona Díaz L., Porta Sales J. (2011). Frequency of pain as a reason for visiting a primary care clinic and its influence on sleep. Aten. Primaria.

[5] Buttagat V., Narktro T., Onsrira K., Pobsamai C. (2016). Short-Term Effects of Traditional Thai Massage on Electromyogram, Muscle Tension and Pain among Patients with Upper Back Pain Associated with Myofascial Trigger Points. Complement. Ther. Med..

[6] Ganesh G.S., Singh H., Mushtaq S., Mohanty P., Pattnaik M. (2016). Effect of Cervical Mobilization and Ischemic Compression Therapy on Contralateral Cervical Side Flexion and Pressure Pain Threshold in Latent Upper Trapezius Trigger Points. J. Bodyw. Mov. Ther..

[7] María Loreto Díaz J. (2014). Cervicalgia Miofascial. Rev. Medica Clin. Las Condes.

[8] Cagnie B., Dewitte V., Coppieters I., Van Oosterwijck J., Cools A., Danneels L. (2013). Effect of Ischemic Compression on Trigger Points in the Neck and Shoulder Muscles in Office Workers: A Cohort Study. J. Manip. Physiol. Ther..

[9] Fernández-de-Las-Peñas C., Alonso-Blanco C., Fernández-Carnero J. (2006). Page, juan-carlos The Immediate Effect of Ischemic Compression Technique and Transverse Friction Massage on Tenderness of Active and Latent Myofascial Trigger Points: A Pilot Study. J. Bodyw. Mov. Ther..

[10] Gerwin R.D. (2005). Factores que promueven la persistencia de mialgia en el síndrome de dolor miofascial y en la fibromialgia. Fisioterapia.

[11] Daniels J.M., Ishmael T., Wesley R.M. (2003). Managing Myofascial Pain Syndrome: Sorting through the Diagnosis and Honing Treatment. Phys. Sportsmed..

[12] Sun A., Yeo H.G., Kim T.U., Hyun J.K., Kim J.Y. (2014). Radiologic Assessment of Forward Head Posture and Its Relation to Myofascial Pain Syndrome. Ann. Rehabil. Med..

[13] Kang J.-H., Park R.-Y., Lee S.-J., Kim J.-Y., Yoon S.-R., Jung K.-I. (2012). The Effect of the Forward Head Posture on Postural Balance in Long Time Computer Based Worker. Ann. Rehabil. Med..

[14] Pacheco J., Raimundo J., Santos F., Ferreira M., Lopes T., Ramos L., Silva A.G. (2018). Forward Head Posture Is Associated with Pressure Pain Threshold and Neck Pain Duration in University Students with Subclinical Neck Pain. Somatosens. Mot. Res..

[15] Fernández-de-Las-Peñas C., Cook C., Cleland J.A., Florencio L.L. (2023). The Cervical Spine in Tension Type Headache. Musculoskelet. Sci. Pract..

[16] Travell J.G., Simons D.G. (1999). Myofascial Pain and Dysfunction. 1: Upper Half of Body.

[17] Garson J.G. (1885). The Frankfort Craniometric Agreement, with Critical Remarks Thereon. J. Anthropol. Inst. G. B. Irel..

[18] Capon T. (2016). Standardised Anatomical Alignment of the Head in a Clinical Photography Studio. A Comparison between the Frankfort Horizontal and the Natural Head Position. J. Vis. Commun. Med..

[19] Carrasco-Bustos J., Freundlich-Deutsch T., Peñafiel-Ekdhal C., Estay-Larenas J., Vergara-Núñez C., Carrasco-Bustos J., Freundlich-Deutsch T., Peñafiel-Ekdhal C., Estay-Larenas J., Vergara-Núñez C. (2019). Relación Entre La Posición Natural de Cabeza y El Plano de Frankfort. Rev. Clin. Periodoncia Implantol. Rehabil. Oral.

[20] Rodríguez Romero B., Mesa Jiménez J., Paseiro Ares G., González Doniz M. (2004). Síndromes posturales y reeducación postural en los trastornostemporomandibulares. Rev. Iberoam. Fisioter. Kinesiol..

[21] Quiles Mateo A. (2020). Tratamiento postural mediante reequilibrio de cadenas miofasciales. NPunto.

[22] Verma S.K., Maheshwari S., Gautam S.N., Prabhat K., Kumar S. (2012). Natural Head Position: Key Position for Radiographic and Photographic Analysis and Research of Craniofacial Complex. J. Oral Biol. Craniofacial Res..

[23] Solow B., Tallgren A. (1971). Natural Head Position in Standing Subjects. Acta Odontol. Scand..

[24] Carrasco Bustos J. (2017). Estudio Comparativo del Paralelismo Entre la Horizontal Verdadera y Tres Planos Trazados Desde el Pabellón Auricular Hacia el Punto Suborbitario. Master’s Thesis.

[25] Alvial-Vergara L., Linker-Navarro K., Vergara-Núñez C., Alvial-Vergara L., Linker-Navarro K., Vergara-Núñez C. (2021). Posición Natural de Cabeza y Su Relación Con El Plano de Frankfurt En Cefalometría Ortodóncica. Int. J. Interdiscip. Dent..

[26] Devi S.S., Dinesh S., Sivakumar A., Nivethigaa B., Alshehri A., Awadh W., Alam M.K., Bhandi S., Raj A.T., Patil S. (2022). Reliability of Frankfort Horizontal Plane with True Horizontal Plane in Cephalometric Measurements. J. Contemp. Dent. Pract..

[27] Chesterton L.S., Sim J., Wright C.C., Foster N.E. (2007). Interrater Reliability of Algometry in Measuring Pressure Pain Thresholds in Healthy Humans, Using Multiple Raters. Clin. J. Pain.

[28] Kinser A.M., Sands W.A., Stone M.H. (2009). Reliability and Validity of a Pressure Algometer. J. Strength Cond. Res..

[29] Reeves J.L., Jaeger B., Graff-Radford S.B. (1986). Reliability of the Pressure Algometer as a Measure of Myofascial Trigger Point Sensitivity. Pain.

[30] Gerwin R.D., Shannon S., Hong C.Z., Hubbard D., Gevirtz R. (1997). Interrater Reliability in Myofascial Trigger Point Examination. Pain.

[31] Delaney G.A., McKee A.C. (1993). Inter- and Intra-Rater Reliability of the Pressure Threshold Meter in Measurement of Myofascial Trigger Point Sensitivity. Am. J. Phys. Med. Rehabil..

[32] Yap E.-C. (2007). Myofascial Pain—An Overview. Ann. Acad. Med. Singap..

[33] Rubine-Gatina S., Rimere N., Zundane Z., Gulajeva A., Reste J. (2022). Sternocleidomastoid Muscle and Head Position: How to Minimize Muscle Tension. IISE Trans. Occup. Ergon. Hum. Factors.

